# An analysis of risk factors of non-fatal drowning among children in rural areas of Guangdong Province, China: a case-control study

**DOI:** 10.1186/1471-2458-10-156

**Published:** 2010-03-25

**Authors:** Wen Jun Ma, Shao Ping Nie, Hao Feng Xu, Yan Jun Xu, Xiu Ling Song, Qiao Zhi Guo, Yu Run Zhang

**Affiliations:** 1 Department of Non-communicable Disease Control and Prevention, Center for Disease Control and Prevention of Guangdong Province, China

## Abstract

**Background**

Drowning is a major cause of morbidity and mortality for children, yet non-fatal drowning remains poorly understood. The aim of this study was to explore potential modifiable risk factors of non-fatal drowning among children in rural areas of China.

**Methods**

A cross-sectional survey was first conducted to obtain non-fatal drowning cases, and 7432 students in grades three to eight from 17 schools participated in the cross sectional survey. Of these, 805 students reported that they experienced non-fatal drowning in the previous year. Then 368 cases were selected randomly to participate in a 1:1 matched case-control study. Each drowning case was matched by one control with the same sex and similar age (the gap less than 2 years) who was selected randomly from the same class.

**Results**

Boys were more likely to be involved in non-fatal drowning. Non-fatal drowning most often happened in the afternoon (65.1%) and natural bodies of water were the most common sites of drowning (71.1%). Swimming, diving and playing in natural waters were the leading activities that preceded non-fatal drowning. The significant risk factors for non-fatal drowning were swimming in natural waters without adult supervision (OR = 3.40, 95% CI: 1.92-6.03), playing in or beside natural waters (OR = 2.08, 95% CI: 1.17-3.70) and poor swimming skills (OR = 2.74, 95% CI: 1.14-6.62). However, the following variables were protective factors: supervisor aged 30 years or over (OR = 0.20, 95% CI: 0.09-0.49) and no water activities (OR = 0.36, 95% CI: 0.18-0.70).

**Conclusions**

The reduction in dangerous water activities, swimming training and enhancement in supervision among children might decrease the risk of non-fatal drowning.

## Background

Drowning is a leading cause of death for children and adolescents in the world [1-4]. Over half of the global drowning mortality occurs among children less than 15 years of age, and most of these occur in developing countries[5]. As a developing country, China ranked the second highest drowning mortality in the world[5]. World Health Organization(WHO) estimated that approximately 129 000 people died of drowning in China in 2000, accounting for over one quarter of the world's drowning deaths[2]. Children are also a vulnerable population to drowning in China. It was estimated that half of all drowning deaths in China occurred among children under 15 years in 2002[6] and that children in rural areas are at the highest risk of drowning [7-9]. WHO reported the drowning mortality rates for children aged 1-4 years, 5-9 years and 10-14 years were 19.34, 16.82, and 13.98 per 100 000 person years in rural China, respectively. These mortalities were 4.6, 9.1, and 10.1 times that of counterparts in urban areas [5].

Deaths from drowning are only the tip of iceberg compared to non-fatal drowning. Moon's study shows that for every child who drowns, four are hospitalized and 16 receive emergency department care for non-fatal drowning[10]. Another study find that for every 10 children who die of drowning, 140 are treated in emergency rooms and 36 are admitted to hospitals for further treatment[11]. Moreover, many survivors of non-fatal drowning have permanent neurological disabilities. Therefore, non-fatal drowning imposes heavy burdens on individual, family and society. However, there are few population based studies to address non-fatal drowning.

Drowning is a multi-causal event. Many factors have been identified as being involved in fatal drowning incidents in developed countries. They include drinking alcohol prior to aquatic activities, inadequate caregivers supervision, lack of swimming skills and unsafe aquatic environments [4,6,10,12]. However, the risk factors of drowning differ with different countries and regions due to socioeconomic and geographic differences [13-16]. For example, there are few private swimming pools in the rural regions of developing countries. Therefore, lack of swimming pool fence is not a risk factor of drowning. Conversely, lack of safe swimming facilities is a very important risk factor in developing countries.

Although drowning is a very serious public health problem in developing countries, research and prevention strategies have been largely confined to developed countries [2,4,17-19] and as such there is little understanding about drowning in developing countries[20]. Recently, although some cross-sectional studies have described the characteristics of drowning such as place of drowning, time of drowning, person accompanying and swimming ability, there is no successful model for implementing the known, effective interventions from high-income countries in a low-income country[20,21].

China is no exception to this with almost all previous studies focused on description of drowning mortality [6,7,20,22]. In order to understand non-fatal drowning in primary and secondary schools, we conducted a cross-sectional survey to describe the incidence rate and distribution of non-fatal drowning in 2006, which indicated non-fatal drowning was a very serious public health problem for rural students[23]. Therefore, we initiated a further study to evaluate the effect of water safety education on non-fatal drowning in 2007. Before this safety education intervention, one 1:1 matched case-control study was conducted to explore risk factors of non-fatal drowning, which was expected to provide information for intervention. This paper reported the main findings of this case-control study, and sought to explore socioeconomic, environmental, and behavioral risk factors of non-fatal drowning of students in rural areas of Guangdong province, China.

## Methods

### Study site

Guangdong Province is located on the south coast of China. The Province is a water-oriented Province with long and hot summers. The annually average temperature varies 18.7-23.4°C, and summer sustains 6 months, going through May to October(25.6°C,27.3°C,28.4°C,28.3°C,27.0°C,23.9°C, respectively). Natural bodies of water such as rivers, reservoirs, dams and ponds are very common in the province. There are 642 rivers with more than 100 km^2 ^catchment areas for every river, over 6000 reservoirs, and numerous ponds. Lianping County is located in the north-east of Guangdong province. There are 13 townships in the county. It is a mountainous county with many aquatic environments (rivers, ponds and reservoirs). The population of the county is around 380 000. Most residents are farmers with less than high school educational level. The county is economically undeveloped and the annual income per farmer was 3600 RMB in 2006. There is only one public swimming pool in the county, and natural bodies of water are the major places of water activities in hot summer.

### Study subjects

Drowning is defined as the process of experiencing respiratory impairment from submersion/immersion in liquid[24]. Non-fatal drowning means experiencing a drowning event but recovering from it or no morbidity. In order to obtain non-fatal drowning cases, a cross sectional survey was conducted prior to the matched case-control study in 2007. Eight townships were firstly selected randomly from Lianping County. Then one primary school and one secondary school were obtained randomly from each township except for Zhongxin Township where two primary schools and one secondary school were selected. Thirdly, 8114 students from grades three to eight in 17 schools were invited to respond. They were required to finish self-administered questionnaires in the classroom. The number of respondents was 7432, and the response rate reached 91.6% (7432/8114).

Eight hundred and five students reported that they had experienced a non-fatal drowning incident in the previous year of the survey. According to the formula of sample size for 1:1 matched case-control study, at least 300 pair case-controls are estimated for this study. Balancing response rate and research resources, 368 cases were selected randomly to conduct this matched case-control study. A control was defined as a student who did not experience a non-fatal drowning event during the same period, and obtained randomly from the same class as its matched case. Every control has the same sex and a similar age (the gap was less than two years of age) with its matched case.

### Questionnaire development

The case-control study collected data using a constructed questionnaire. The draft questionnaire was developed on the basis of a literature review [16-18,20,25,26]. Then it was reviewed and refined by experts from injury prevention and control. Finally, a pilot study was carried out in a primary school to test and refine the questionnaire. The questionnaire consisted of demographic characteristics, student behaviors related to drowning, safety education, caregivers' supervision and environmental features. Demographic characteristics included student age, gender, personality (extrovert or introvert), parental education and household income. Variables relating to student's behaviors included swimming ability and risk behaviors (swimming in natural waters without supervision, fishing alone in natural bodies of water, playing/diving in or beside natural water, whether using life vest during water activities). Safety education and supervision variables included safety education in school, supervisor, supervisor's age, and supervisor's first aid skills. Risk environment variables included warning signs around natural waters, drinking-water well lids, fencing around natural waters.

### Data collection and analysis

The data of both cases and controls were collected by face to face interview. All interviewers were from the Center for Disease Control and prevention of Lianping County, and they were trained by the research team. However, the interviewers were not blinded when they collected data. The study protocol was approved by the ethic committee of Center for Disease Control and Prevention of Guangdong province. All respondents signed an informed consent form before they participated in the survey. Name was not recorded on the questionnaire to protect respondents' privacy.

All questionnaires were doubly entered using software Epidata3.0 to reduce entry error. Statistical analysis was performed by SAS9.13 software. Descriptive analysis was conducted to describe the drowning characteristics and basic information of the sample. A two step approach was used to analyze risk factors of non-fatal drowning. First, bivariate model was fitted for each independent variable and dependent variable. Independent variables included children's personality, mother's education, father's education, household income, swimming skills, water safety education, environmental factors, swimming/fishing/playing/diving in natural waters, whether using life vest as recreational water activity, who being as supervisor, supervisor's age, supervisor's first aid skills and neighbor's first aid skills. Then, additional adjustment of the effect of each risk factor for all other statistically significant factors was performed in a multivariate conditional logistic regression models. Odds ratios (OR) with the corresponding 95% confidence intervals (95% CI) were calculated, but only significant or near significant results are presented in the paper (significant level α = 0.05).

## Results

### Characteristics of the sample

Three hundred and sixty eight matched case-controls were initially recruited into the study. Forty-three case-controls were not included in the analysis due to too much incomplete information. Therefore, 325 matched case-controls were analyzed. χ^2 ^test showed that there were no significant differences in demographics and socioeconomic status between the 43 excluded cases and the 325 included cases. Table 1 showed that cases and controls had similar demographic and socioeconomic status.

**Table 1:** Comparison of demographics and socioeconomic status between cases and controls

	Cases n(%)	Controls n(%)	P-value (χ^2 ^test)
Age(years)	325(100.0)	325(100.0)	0.545
9-11	35(10.7)	38(11.7)	
12	54(16.6)	58(17.8)	
13	48(14.8)	32(9.8)	
14	83(25.5)	97(29.8)	
15	75(23.1)	71(21.8)	
16-18	30(9.2)	29(8.9)	
Father's education	325(100.0)	325(100.0)	0.605
Illiteracy	4(1.2)	4(1.2)	
Elementary school	39(12.0)	51(15.7)	
High school	140(43.1)	148(45.5)	
Undergraduate	54(16.6)	51(15.7)	
Postgraduate and over	21(6.5)	14(4.3)	
Unknown	67(20.6)	57(17.5)	
Mother's education	252(100.0)	255(100.0)	0.558
Illiteracy	9(2.8)	6(1.9)	
Elementary school	88(27.1)	92(28.4)	
High school	101(31.1)	109(33.6)	
Undergraduate	39(12.0)	40(12.4)	
Postgraduate and over	15(4.6)	8(2.5)	
Unknown	73(22.5)	69(21.3)	
Household income	325(100.0)	325(100.0)	0.344
Low	33(10.2)	23(7.1)	
In-betweens	228(70.2)	223(68.8)	
High	52(16.0)	65(20.1)	
Unknown	12(3.7)	13(4.0)	

### Characteristics of non-fatal drowning

Almost two thirds of the cases were boys. Sixty five percent of all cases occurred in the afternoon, 27% at noon, and 7% in the morning. Natural bodies of water were the most common non-fatal drowning sites. Seventy five percent of all cases occurred in natural waters (rivers/ponds/water ditches/cisterns), 15% in swimming pools and 2% in drinking-water wells. Most incidents (80%) occurred in the warm months (May 26%, June 36%, July 17%). Swimming, diving and playing in natural waters were the leading activities prior to drowning, attributing 42%, 5%, and 11%, respectively. Non-fatal drowning was mainly caused by falling into water accidentally (25%), swimming (24%), diving (17%), playing in water (13%). Most of victims (76%) reported that they were supervised by caregivers when they sustained non-fatal drowning. Eighty three percent of cases reported that there were natural waters around schools or homes, and 76% of these natural waters did not have warning signs. Thirty cases (9.4%) reported that they lost consciousness when they are rescued, and only 4.7% of all cases were sent to treat in hospital.

### Bivariate analysis of risk factors of drowning

Table 2 provides a summary of the statistically significant results of bivariate logistic regression analysis. The findings revealed that poor swimming skills, swimming/fishing in natural bodies of water in the absence of adult supervision, playing/diving in natural waters, supervision by old siblings/neighbors/relatives were positively associated with increased risk of sustaining non-fatal drowning. Factors associated with a decreased risk of non-fatal drowning included no natural bodies of water next to schools or homes, no recreational water activities in the past year, incapable of swimming, usually supervision by mother, supervisor's age above 30 years or no supervisor (compared to supervisor less than 20 years).

**Table 2:** Biivariate analysis of risk factors for non-fatal drowning in rural areas, Guangdong, China

Factors		Odds ratio	95% CI
Children's personality	Extrovert	1.00	reference
	In-betweens	0.99	0.62-1.59
	Introvert	0.94	0.48-1.85
Father's education	Illiteracy	1.00	reference
	Elementary school	1.14	0.18-7.30
	High school	1.51	0.24- 9.70
	Undergraduate	1.43	0.23- 8.80
	Postgraduate and over	2.34	0.34-15.97
Mother's education	Illiteracy	1.00	reference
	Elementary school	0.62	0.20-1.95
	High school	0.58	0.18-1.86
	Undergraduate	0.52	0.15-1.75
	Postgraduate and over	1.22	0.25-5.95
Water safety education in school	No	1.00	reference
	Yes	1.11	0.74-1.67
	Can't remember	1.11	0.63-1.94
Household income	*High*	1.00	reference
	*Low*	1.99	1.01-3.95
warning sign around natural bodies of water	*Yes*	1.00	reference
	*No natural bodies of water*	0.54	0.31-0.91
Swimming ability	*Good*	1.00	reference
	*Incapable of swimming*	0.52	0.27-0.98
	*Poor*	3.30	1.69-6.43
Swimming in natural bodies of water without adult supervision in last 12 months	*No*	1.00	reference
	*Yes*	3.74	2.56-5.45
Fishing in natural waters in absence of adult supervision in last 12 months	*No*	1.00	reference
	*Yes*	1.58	1.09-2.29
Playing in natural waters in last 12 months	*No*	1.00	reference
	*Yes*	2.71	1.83-4.01
Diving in natural waters in last 12 months	*No*	1.00	reference
	*Yes*	2.81	1.88-4.21
Using life vest in water in last 12 months	*Yes*	1.00	reference
	*No recreational water activity*	0.27	0.16-0.45
Supervisor	*father*	1.00	reference
	*mother*	0.56	0.36-0.86
	*Siblings/neighbors/relatives*	2.91	1.31-6.43
Supervisor's age	*<20*	1.00	reference
	*≥ 30*	0.11	0.04-0.28
	*No supervisor*	0.18	0.07-0.46

### Multivariate analysis of risk factors of drowning

In order to adjust for confounding factors, a backward stepwise multiple conditional logistic regression was employed using all the above statistically significant variables. The results are displayed in Table 3. It can be seen that the following factors were no longer associated with non-fatal drowning when adjusting confounding factors: low household income, natural bodies of water next to school or home, incapability to swim, fishing/diving in natural bodies of water, mother as supervisor, old siblings/neighbors/relatives as supervisors. Other exposure variables that significantly contributed to the bivariate models were included in multivariate analysis. They were (1) swimming/fishing in natural bodies of water without adult supervision, (2) playing in natural bodies of water, (3) poor swimming ability, (4) no water activities in last 12 months, (5) supervisor's age above 30 years or no supervisor. Out of them, the first three variables were risk factors of non-fatal drowning, and the latter two variables were protective factors.

**Table 3:** Multivariate analysis of risk factors for non-fatal drowning in rural areas, Guangdong, China

Risk factors	Odds Ratio	95% CI
Swimming ability		
*Poor*	2.74	1.14-6.62
*good*	1.00	reference
Swimming/fishing in natural waters in absence of adult supervision in last 12 months		
*Yes*	3.40	1.92-6.03
*no*	1.00	reference
Playing in natural waters in last 12 months		
*yes*	2.08	1.17-3.70
*no*	1.00	reference
Using life vest in water activities in last 12 months		
*No recreational water activity*	0.36	0.18-0.70
*yes*	1.00	reference
Supervisor's age(year)		
*≥ 30*	0.20	0.09-0.49
*No supervisor*	0.36	0.14-0.91
*<20*	1.00	reference

## Discussion

Most previous studies on drowning focused on description of drowning mortality and trends [13,27-29]. There no population-based studies were found to characterize non-fatal drowning and explore its risk factors in China. To fill this gap, the authors conducted a cross-sectional survey to describe the incidence rate of non-fatal drowning in 2006, which suggested non-fatal drowning posed great threats to rural students[23]. This indicated it was very necessary to initiate further study to address drowning problem in rural areas of China. Thus, the authors carried out this case-control study as one part of a water safety education program to provide knowledge for intervention in 2007. The findings of this study revealed that boys were at higher risk of non-fatal drowning compared with girls, which suggests that gender is an important risk factor regardless of the severity of drowning [4,6,19]. Most non-fatal drowning incidents happened in natural bodies of water, which is similar with fatal drowning data in China reported by Yang and Fang[6,20], but it is different from non-fatal drowning in U.S. where 75% of non-fatal drowning occurred in swimming pools in 2002 [13]. This indicates that the locations of non-fatal drowning are likely to be different between and within countries due to geographical and economic differences. The warmer season and the afternoon period were the most at-risk times for children to be involved in non-fatal drowning incidents. This phenomenon was observed in other studies in both developing and developed countries [6,9,13,20]. The likely explanation is that hot weather increases the demand for water activities and lack of adult supervision after school in the afternoon contributes to this at-risk time. If these converge with aquatic environments located near school or home, the risk of a drowning incident may significantly increase.

The study found that risk behaviors, such as swimming in natural waters without adult supervision and playing in or around natural waters, significantly increased children's risk of non-fatal drowning. The possible explanation is the convergence of increasing exposure and underdeveloped cognition of children, which puts children at high risk for drowning. Due to cognitive development immaturity, children have low risk perception and are more likely to exhibit more impulsive and daring behaviors in water, such as playing and diving in water. In fact, it is evident that exposure level is a very important determinant for drowning[30,31]. This study also found that children not partaking in recreational water activities in last 12 months were less likely to occur non-fatal drowning. In addition, it could be seen in this study that recreational water activities such as swimming, diving, and playing in water accounted for more than two thirds of activities immediately prior to drowning. This implicated that reduction of exposure level to dangerous bodies of water might have potential for drowning prevention. Public swimming pools are safer places for children's water activities than natural bodies of water. However, it is impossible to build many public swimming pools to meet children's needs for water activities due to undeveloped economic situation in rural areas in China. Therefore, building simple swimming facilities around natural bodies of water may be a realistic choice, but it is merited to pay attention to the problems such as safety and management arising from these man-made facilities.

Some studies have showed that swimming training can improve children's swimming ability and safety skills [32,33]. Meanwhile, some descriptive and ecological studies have suggested that swimming ability is an important protective factor in reducing children drowning incidents [33,34]. However, the effectiveness of swimming ability in reducing risk of drowning has never been clearly demonstrated. Conversely, some researchers raise concerns about swimming training because increased swimming ability might increase exposure to hazardous water environment and decrease parent's vigilance and supervision when children are swimming in water [31,34,35]. Indeed, Howland found that males who reported better swimming ability than females, also reported greater exposure to water as well as high risk behaviors such as swimming alone and swimming in natural bodies of water [36]. The present study also found that children with better swimming ability had more risk behaviors such as swimming, playing, fishing, and diving in natural bodies of water (not shown in the results). However, after adjustment for these risk exposures and other confounding factors, poor swimming ability remained significantly associated with an increased risk of non-fatal drowning. These results correspond with two previous case-control studies conducted in U.S.A [33,37]. This finding has significant potential for drowning prevention because swimming training can reduce risk of drowning in different situations for various age populations rather than only targeting some specific environment and age groups (eg. swimming pool fencing).

Adult supervision for children in drowning prevention was emphasized by many studies. For example, Laden and Quan found that drowning typically involved a supervision standard violation [38,39]. However, supervision is a complex and multi-faceted phenomenon. It is important to consider the interaction between the type of supervisor, supervision pattern and level, child and environment. One study showed mother and father had different supervision patterns, and fathers was more likely to intervene their children than mothers under certain conditions [40]. Meanwhile, some research suggested that older siblings as supervisors increased risk of injury [41]. This study's findings confirmed these previous studies. The bivariate analysis revealed that the mother as a supervisor was a protective factor for non-fatal drowning, and sibling/neighbor/relative as a supervisor was a risk factor for non-fatal drowning. None of these factors remained statistically significant after controlling for other factors in the multivariate analysis. However, it is cautious to explain this finding because this study just collected data about 'who was usual supervisor' rather than data about supervisors during near-drowning. This limitation may reduce association between inadequate supervision and non-fatal drowning because usual supervisors might not present during a non-fatal drowning event. Despite the lack of statistically significant findings in the multivariate analysis, inadequate supervision is more likely to play an important role in drowning injury in rural areas of China because many farmers have migrated to the cities for employment and have left their children behind at home. An unintentional finding was that a younger supervisor (less than 20 years of age) significantly increased the risk of non-fatal drowning compared to no supervisor. A possible explanation for this is that most of younger supervisors may be older siblings who have a negative impact on risk behaviors of their younger siblings[41]. Although this study did not collect data about person accompanying as drowning, a recent study from Bangladesh found that in drowning cases in which child was accompanied almost half were children who were 10 years or below[21].

Many studies have noted that there is a gradient relationship between drowning and socioeconomic status[6,17,42]. The study didn't find low household income was associated with increased risk of non-fatal drowning. Potential reason is the subjects of the study came from the similar socioeconomic families, which is not suitable for studying socioeconomic status. The explanation might be similar for environmental factors not being confirmed in this study because controls and cases lived in the similar environments.

Several limitations of this study should be considered in the interpretation of the results. First, like any retrospective study, recall bias is unavoidable and potentially affects the estimates. Another limitation is that some information such as personality and family income might be inaccurate because it was collected by self-reporting. Thirdly, although the definition of drowning was well described in the questionnaire, the dividing line between underwater fun and a mild non-fatal drowning incident is not clear. It is possible that some children reported underwater fun as non-fatal drowning leading to over reporting. In the future research, in order to reduce this bias, it might be meaningful to restrict the cases to those who had a drowning event that required treatment, or that resulted in loss of consciousness, or that required CPR treatment. Nevertheless, this study about non-fatal drowning has provided important information to conduct more advanced research in China.

## Conclusions

This study found that non-fatal drowning had the similar risk factors with fatal drowning in China. Water exposures, poor swimming abilities and inadequate supervision were major risk factors for non-fatal drowning. The results suggest that reduction in dangerous water activities, swimming training and enhancement in supervision among children might decrease the risk of non-fatal drowning.

## Competing interests

The authors declare that they have no competing interests.

## Authors' contributions

MWJ was responsible for project design, statistical analysis and writing of the manuscript. NSP, XHF, XYJ, SXL, GQZ and ZYR participated in the project design and implemented the study. All authors read and approved the final manuscript.

## Pre-publication history

The pre-publication history for this paper can be accessed here:

http://www.biomedcentral.com/1471-2458/10/156/prepub
